# Resting state connectivity predictors of symptom change during gaze-contingent music reward therapy of social anxiety disorder

**DOI:** 10.1017/S0033291721005171

**Published:** 2023-05

**Authors:** Xi Zhu, Amit Lazarov, Sarah Dolan, Yair Bar-Haim, Daniel G Dillon, Diego A Pizzagalli, Franklin Schneier

**Affiliations:** 1 Department of Psychiatry, Columbia University Irving Medical Center, New York, USA; 2 New York State Psychiatric Institute, New York, USA; 3 School of Psychological Sciences, Tel-Aviv University, Tel-Aviv, Israel; 4 Department of Psychiatry, McLean Hospital/Harvard Medical School, Belmont, MA, USA

**Keywords:** Attention bias modification, gaze-contingent music reward therapy, neuroimaging, resting state fMRI, social anxiety disorder

## Abstract

**Background:**

Social anxiety disorder (SAD) is common, first-line treatments are often only partially effective, and reliable predictors of treatment response are lacking. Here, we assessed resting state functional connectivity (rsFC) at pre-treatment and during early treatment as a potential predictor of response to a novel attention bias modification procedure, gaze-contingent music reward therapy (GC-MRT).

**Methods:**

Thirty-two adults with SAD were treated with GC-MRT. rsFC was assessed with multi-voxel pattern analysis of fMRI at pre-treatment and after 2–3 weeks. For comparison, 20 healthy control (HC) participants without treatment were assessed twice for rsFC over the same time period. All SAD participants underwent clinical evaluation at pre-treatment, early-treatment (week 2–3), and post-treatment.

**Results:**

SAD and depressive symptoms improved significantly from pre-treatment to post-treatment. After 2–3 weeks of treatment, decreased connectivity between the executive control network (ECN) and salience network (SN), and increased connectivity within the ECN predicted improvement in SAD and depressive symptoms at week 8. Increased connectivity between the ECN and default mode network (DMN) predicted greater improvement in SAD but not depressive symptoms at week 8. Connectivity within the DMN decreased significantly after 2–3 weeks of treatment in the SAD group, while no changes were found in HC over the same time interval.

**Conclusion:**

We identified early changes in rsFC during a course of GC-MRT for SAD that predicted symptom change. Connectivity changes within the ECN, ECN-DMN, and ECN-SN may be related to mechanisms underlying the clinical effects of GC-MRT and warrant further study in controlled trials.

## Introduction

Social anxiety disorder (SAD) is one of the most common psychiatric disorders, with a 5–12% lifetime prevalence and high chronicity and impairment (Kessler et al., 2005; Schneier et al., 1994). Current first-line treatments, such as cognitive behavioral therapy (CBT) and serotonin reuptake inhibitors (SSRIs), are only partially effective for most patients (Schneier, 2011; Springer, Levy, & Tolin, 2018), highlighting the need for new interventions addressing novel treatment targets. Biased attention allocation is a characteristic of SAD that has emerged as a promising target of treatment. Among individuals with SAD, visual attention is biased toward socially threatening stimuli, and some controlled trials have shown that, systematic modification of this bias can reduce SAD symptoms (Heeren, Mogoase, Philippot, & McNally, 2015; Lazarov et al., 2018; Lazarov, Pine, & Bar-Haim, 2017).

Gaze-contingent music reward therapy (GC-MRT) is a novel computer-based attention bias modification procedure. It tracks eye gaze as participants view matrices of 16 faces, half with neutral and half with threatening (i.e. disgust) expressions. Participants' chosen music plays only when gaze is directed at neutral faces but stops when attention is allocated to any of the threat faces (Lazarov et al., 2017). Through this musical reinforcement, participants gradually learn to preferentially allocate their attention to the non-threat (i.e. neutral) faces. In a randomized controlled trial, eight sessions of GC-MRT delivered over a period of 4 weeks elicited greater clinical improvement than a control condition (Lazarov et al., 2017), and changes in attention allocation were observed beginning with the first week of treatment.

While GC-MRT has been shown to modulate attention to threat assessed at the level of gaze behavior (Lazarov et al., 2017; Shamai-Leshem, Lazarov, Pine, & Bar-Haim, 2021), the neural mechanisms underlying GC-MRT effects remain to be explored. The goal of the present study was to address this gap in knowledge by assessing resting state functional connectivity (rsFC) of neural networks before and at an early stage of GC-MRT in patients with SAD. Prior studies of resting state functional magnetic resonance imaging (rs-fMRI) in SAD have identified abnormalities in networks that underlie threat processing (Prater, Hosanagar, Klumpp, Angstadt, & Phan, 2013), social cognition (Choi, Shin, Ku, & Kim, 2016), and attention (Liao et al., 2010a) including the executive control network (ECN), the salience network (SN), and the default mode network (DMN). The ECN is engaged by high-level cognitive functions and consists of middle frontal gyrus (MFG), superior frontal gyrus (SFG), inferior frontal gyrus, orbitofrontal cortex (OFC), inferior (ITG) and middle temporal gyri, and superior and inferior parietal lobe (Shirer, Ryali, Rykhlevskaia, Menon, & Greicius, 2012). Decreased connectivity within the ECN has been reported in SAD and depression and may underlie deficits in cognition and attention that are commonly observed in SAD and depression (Ding et al., 2011), contributing to symptoms such as difficulty concentrating or regulating emotions (Zhao, Swati, Metmer, Sang, & Lu, 2019). The SN, consisting mainly of the amygdala, insula, and dorsal anterior cingulate cortex, plays a central role in detecting salience, either internally or externally-generated, and directing attention allocation and cognitive response via its functional connectivity with other distributed networks (Menon & Uddin, 2010). The SN may also play a critical role in orchestrating the toggling between ECN and DMN to allocate attentional resources toward whichever network is needed, and in signaling salience to the ECN for cognitive control (Sylvester et al., 2012). A body of research also suggests that anxiety and depressive disorders, including SAD, are characterized by altered rsFC within the DMN (Coutinho et al., 2016; Hamilton et al., 2011; Lucherini Angeletti, Scalabrini, Ricca, & Northoff, 2021; Zhou et al., 2010). The DMN, consisting of parahippocampus/hippocampus (HIP), posterior cingulate cortex (PCC), precuneus, and medial prefrontal cortex, has been implicated in self-referential processing (Qin & Northoff, 2011), mind-wandering (Fox, Spreng, Ellamil, Andrews-Hanna, & Christoff, 2015), and memory (Ward et al., 2014).

No studies to date have explored the relation between rsFC and attention bias modification (ABM) treatment outcomes in SAD, although one study has assessed rsFC measuring low-frequency fluctuations (ALFF) in subthreshold depression participants (Li et al., 2016). Findings revealed that ABM treatment significantly reduced the amplitude of ALFF of the right anterior insula and the right MFG and between the right anterior insula and the right insula. In addition, three studies have examined rsFC using an amygdala seed as a predictor or correlate of CBT response, with mixed results (Klumpp, Keutmann, Fitzgerald, Shankman, & Phan, 2014; Whitfield-Gabrieli et al., 2016; Yuan et al., 2016). Yet, a seed-based approach may miss meaningful rsFC changes not hypothesized *a priori*. Multi-voxel pattern analysis (MVPA) has gained traction in recent years as a data-driven approach to map spatially distributed patterns of brain activation and/or rsFC. This approach examines whole-brain distributed neural activation patterns, leveraging the fact that brain function consists of spatially distributed processes (Davis et al., 2014). MVPA was used as an additional assessment of pre-treatment rsFC in the third study above (Whitfield-Gabrieli et al., 2016), which found that MVPA results were the strongest predictor of CBT outcome in a model that included seed-based rsFC, whole-brain MVPA rsFC, diffusion-weighted MRI fractional anisotropy (FA), and pretreatment clinical severity ratings.

The present study used MVPA with rs-fMRI at pre-treatment and after 2–3 weeks of treatment to detect early neural changes that might predict clinical symptom changes after treatment completion (after 8 weeks). This was part of a larger clinical trial designed as an initial step toward clarifying the mechanisms of GC-MRT and optimizing its duration. GC-MRT in the original study (Lazarov et al., 2017) included eight sessions delivered over 4 weeks (i.e. two sessions per week); in the current study, adults with SAD were treated with either a 4-week (eight sessions) or 8-week (12 sessions) course of GC-MRT treatment (online Supplementary Fig. S2). Healthy control (HC) participants were also assessed twice for rsFC over a 2–3 week time interval but did not receive GC-MRT. The present study had three goals. First, we examined whether changes in rsFC from pre-treatment to week 2–3 were associated with changes in clinical features that might be associated with GC-MRT response – namely, severity of social anxiety, depression, anhedonia, and attention control at week 2–3 and at week 8 (when clinical symptom change was expected to be maximal). We hypothesized that in the SAD group, the early change in rsFC within ECN, DMN, and between ECN-SN would be associated with clinical changes. Second, we compared changes in rsFC from pre-treatment to week 2–3 in SAD to changes in the untreated HC group over the same 2–3 week interval. We expected that in the untreated HC group, there would be no significant changes in rsFC over a 2–3 week interval, and so predicted that rsFC changes would be greater in the SAD group. Finally, we also explored pre-treatment rsFC predictors of response to GC-MRT, as measured by SAD symptoms and other clinical features at week 8.

## Methods

### Participants

The sample included 44 patients with SAD and 20 HC. All participants underwent a clinical evaluation and an initial resting state MRI scan. Thirty-three SAD and 20 HC participants returned for a second scan 2–3 weeks after the first [the 33 SAD completers had a mean of 4.9 sessions (std: 1.1) over 18.2 days (std: 5.5), the 20 HC had a mean of 18 days (std: 5.8) before they had the second MRI scan]. Eleven SAD participants completed only the pre-treatment MRI scan: five did not complete a second scan due to scheduling difficulties, and six were dropped due to COVID-19 travel restrictions (online Supplementary Fig. S1). Tables 1 and 2 summarize demographic information and clinical outcomes of SAD treatment completers and HC. Among the 33 SAD participants, four were in stable pre-existing treatment [supportive psychotherapy (*N* = 1); SSRI pharmacotherapy (*N* = 3)]. The New York State Psychiatric Institute Institutional Review Board approved the study, and participants provided written informed consent.

**Table 1.** Demographic table for SAD and HC

	SAD	HC
*N*	33	20
Age	28.06 (6.57)	28.65 (6.18)
Gender (F/M)	21/12	12/8
Race	White 14 (42.4%)	White 10 (50%)
	Black 6 (18.2%)	Black 6 (30%)
	Asian 6 (18.2%)	Asian 4 (20%)
	Others 7 (21.2%)	Others 0 (0%)

**Table 2.** Descriptive statistics and paired *t* tests of clinical outcomes at pre-treatment, early-treatment (week 2–3), and week 8

	Pre-treatment	Early-treatment	Week 8	Pre–early		Pre-week 8	
	Mean (s.d.)	Mean (s.d.)	Mean (s.d.)	*t*	*p*	*t*	*p*
LSAS	86.06 (15.39)	75.96 (22.64)	63.24 (28.71)	2.82	0.01	4.21	<0.0001
HAM-D	6.18 (4.81)	4.27 (3.66)	4.33 (4.11)	2.50	0.019	2.20	0.035
SHAPS	24.82 (5.55)	23.94 (5.25)	23.42 (6.10)	1.53	0.14	1.39	0.18
ACS	44.76 (5.91)	N.A.	47.68 (7.94)	NA	N.A.	−1.46	0.16
RSAS	17.03 (5.96)	16.41 (7.74)	15.03 (8.21)	0.87	0.39	2.42	0.021

LSAS, Liebowitz Social Anxiety Scale; HAM-D, Hamilton Rating Scale for Depression; SHAPS, Snaith-Hamilton Pleasure Scale; ACS, Attention Control Scale (not assessed at during treatment); RSAS, Revised Social Anhedonia Scale.

Inclusion criteria for SAD participants were: (a) primary DSM-5 diagnosis of SAD; (b) Liebowitz Social Anxiety Scale (LSAS) score ⩾50; (c) 18–60 years old; (d) fluent English; and (e) normal or corrected-to-normal vision. Key exclusion criteria included a current severe depression indicated by a Hamilton Rating Scale for Depression (HAM-D) 17-item score >20 (Hamilton, 1960), or use of any psychotropic medication in the past month, other than a SSRI, serotonin-norepinephrine reuptake inhibitor, or zolpidem for sleep, that had been taken at a stable dose for at least 3 months (see online Supplementary Material for full criteria). HC participants were age- and sex-matched, and did not have any lifetime psychiatric disorders or current psychiatric medication use. All participants were right-handed.

### General procedure

MRI procedures were part of a broader clinical trial assessing mechanisms of GC-MRT and comparing the previously established 4-week (eight sessions) course of treatment (Lazarov et al., 2017) to a course augmented by four additional weekly sessions from week 5 to 8 (online Supplementary Fig. S2). Hence, SAD participants were randomized to the treatment duration of either 4 or 8 weeks, with randomization stratified by presence or absence of pre-existing medication treatment. All participants underwent clinical evaluation at pre-treatment, early-treatment (week 2–3), and week 8, with measures of social anxiety, attention control, depression, and anhedonia. Depression and anhedonia were assessed due to their potential impact on the GC-MRT process of positive reinforcement. In this study, the two groups (4- *v.* 8-week) did not differ in symptomatic improvement after 8 weeks (change in LSAS total score at week 8 was −23.68 for the 8-week group and −22.38 for the 4-week group; *t* = −0.13, df = 33, *p* = 0.901), so the groups were pooled for imaging analyses.

### GC-MRT

**Fig. 1.**
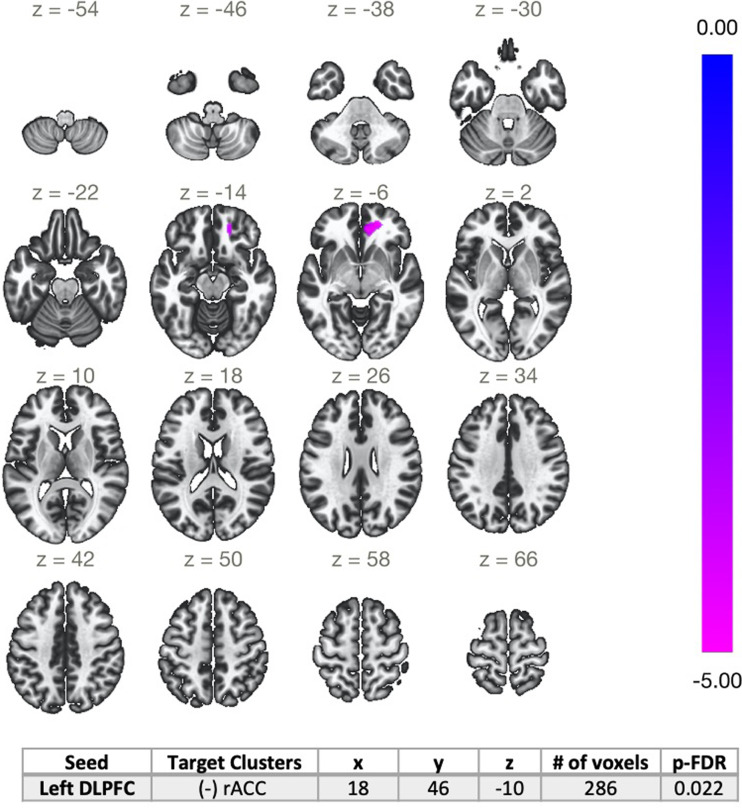
Seed-based whole-brain correlation analysis of changes in rsFC at week 2–3 that were associated with changes in LSAS after week 8 (pre-treatment – week 8). Left DLPFC was used as the seed. Early decreases in rsFC between the left DLPFC-rACC predicted greater improvement in social anxiety at week 8. rsFC, resting state functional connectivity; LSAS, Liebowitz Social Anxiety Scale; DLPFC, dorsolateral prefrontal cortex; rACC, rostral anterior cingulate cortex [18, 46, −10].

The eye-tracking attention modification task was identical to the one used in our previous study in SAD (Lazarov et al., 2017). During each session of GC-MRT, 30 consecutive 4 × 4 matrices, each comprising 16 faces (one from each actor), eight with a disgusted facial expression (i.e. socially threatening stimuli) and eight with a neutral one (i.e. neutral stimuli), were presented to participants, with each matrix presented for 24 s (see Fig. 1 for an example of a single matrix). Before the training session started, patients were asked to choose music that would be used during the session (and after each training session patients rated on a 0–10 scale how much they liked the music played at that session). Next, a five-point gaze calibration, followed by five-point validation, was completed. Calibration was repeated if visual deviation was >0.5 degrees on the *X* or *Y*-axis of any of the calibration points, and training began only when calibration parameters were achieved. After calibration and validation, the GC-MRT session started, during which the chosen music played contingent on the participants' gaze – when the participant fixated on one of the neutral faces the music played, but fixating on any of the threat faces stopped the music. Patients were asked to view each face matrix freely in any way they chose, without additional instructions. Each GC-MRT session was approximately 20 min long and used the Eye-Link 1000 plus eye-tracker running Experiment Builder software (SR Research Ltd., Mississauga, Ontario, Canada). Operating distance to the eye-tracking monitor was about 70 cm. The stimuli were presented on a monitor with 1920 × 1080 pixel resolution.

### Neuroimaging procedures

#### Neuroimaging data acquisition

MRI data were acquired just prior to the first treatment session, and again after 2–3 weeks of treatment. Thirteen participants were scanned using a 3 T General Electric MR750, and 20 participants were scanned using a 3 T General Electric PREMIER (GE Medical Systems, Waukesha, WI, USA) due to a scanner upgrade that occurred during the trial. A harmonized imaging scanning protocol was used for both scanners. The scanner (indicated as 0/1) was included as nuisance regressors in all the rs-fMRI analysis. For each participant, both sessions used the same scanner. A 32-channel receive-only head coil was used. For each participant, a high-resolution T1-weighted 3D BRAVO sequence was acquired using the following parameters: TR = 450 mm, flip angle = 12°, field of view = 25.6 cm, 256 × 256 matrix, slice thickness = 1 mm. Ten-minute eyes-open resting state scans were acquired with TR = 2 s, TE = 30 msec, FA = 77°, FOV = 22 cm, slice thickness = 4 mm, number of volumes = 300.

#### Functional connectivity analysis

Imaging preprocessing is described in the online Supplementary Material. We applied MVPA for model-free voxel-wise rsFC analysis using the CONN toolbox (Whitfield-Gabrieli & Nieto-Castanon, 2012). We used this approach to (1) identify regions/seeds where change in rsFC was associated with change in a specific clinical outcome measure in SAD from pre-treatment to week 2–3, or pre-treatment to week 8, and (2) identify regions/seeds of changes in rsFC from pre-treatment to week 2–3 in patients with SAD, within HC only, and in SAD *v.* HC. Of the five prespecified clinical measures, only those that changed significantly from pre-treatment to week 2–3 or week 8 were analyzed [i.e. LSAS, HAM-D, and Revised Social Anhedonia Scale (RSAS)]. For each subject and session, the MVPA method computed pair-wise correlations between each voxel to all other voxels. Principal components analysis (PCA) was used to reduce data dimensionality (default number of 64 components) by projecting the data from high dimensional space to lower dimensional subspace (principal subspace) such that the variance of the projected data was maximized. The four strongest spatial principal components were selected based on an approximate 5:1 ratio between observations (*N* = 20 for HC, *N* = 33 for SAD) and independent variables (Byun et al., 2021; Thompson, Thelin, Lilja, Bellander, & Fransson, 2016). For a given voxel, *vi*, a PCA was performed by retaining four components, across all subjects, between *vi*'s connectivity with all other voxels. In other words, each voxel had a four-dimensional representation of the spatial pattern of its connectivity to all other voxels for each subject. Then, to assess the association between changes in rsFC from pre-treatment to week 2–3 or pre-treatment rsFC with changes in SAD symptoms, an omnibus *F* test was carried out comparing the between-subject variance in relation to clinical outcomes in SAD across all voxels' connectivity patterns in four-dimensional space. Scanner was used as nuisance regressor. This test yielded clusters of voxels (seeds) that displayed a similar between-subject variance of their spatial connectivity. Once seeds were established, the seeds from the MVPA were extracted as region of interest masks and were applied for seed-to-voxel whole-brain analysis to determine whole-brain connectivity patterns that were associated with the clinical outcomes. To assess the changes in rsFC from pre-treatment to week 2–3, a second *F* test was carried out comparing within-subject variance across all voxels' connectivity patterns in four-dimensional space in SAD and HC, controlling for scanner. Post-hoc analyses were performed using these identified clusters of voxels as seeds in standard seed-to-voxel analyses, and testing the association between seed-to-whole-brain connectivity and clinical outcomes or group by session interaction. Clusters surviving a height threshold of *p* < 0.001 and FWE cluster-level threshold of *p* < 0.05 were taken for post-hoc analysis. The same threshold was applied for post-hoc seed-to-whole-brain rsFC analysis.

## Results

### Treatment results

Severity of social anxiety (LSAS), depression (HAM-D), and social anhedonia (RSAS) improved significantly from pre-treatment to week 8 (Table 2). General anhedonia, as assessed by the Snaith-Hamilton Pleasure Scale (SHAPS), and attention control, assessed by the Attention Control Scale (ACS), did not change significantly from pre-treatment to either week 2–3 or week 8. In the absence of changes in SHAPS or ACS scores, we were unable to assess the rsFC associated with the hypothesized changes.

The 33 SAD completers and the 11 SAD participants that did not provide a second scan did not differ in age, sex, or baseline clinical measures (LSAS, HAM-D, RSAS, SHAPS, ACS; *p* > 0.05). The 33 SAD completers had an average of 4.9 sessions (std: 1.1) and an average of 18.2 days (std: 5.5) of treatment before they had the second MRI scan.

Across all GCMRT sessions, the mean rating of how much they liked the music was 7.35 (std: 2.10), confirming that the selected music had reinforcement value, as expected.

#### Early rsFC changes predicting symptom improvement

- In social anxiety symptoms measured by LSAS

**Fig. 2.**
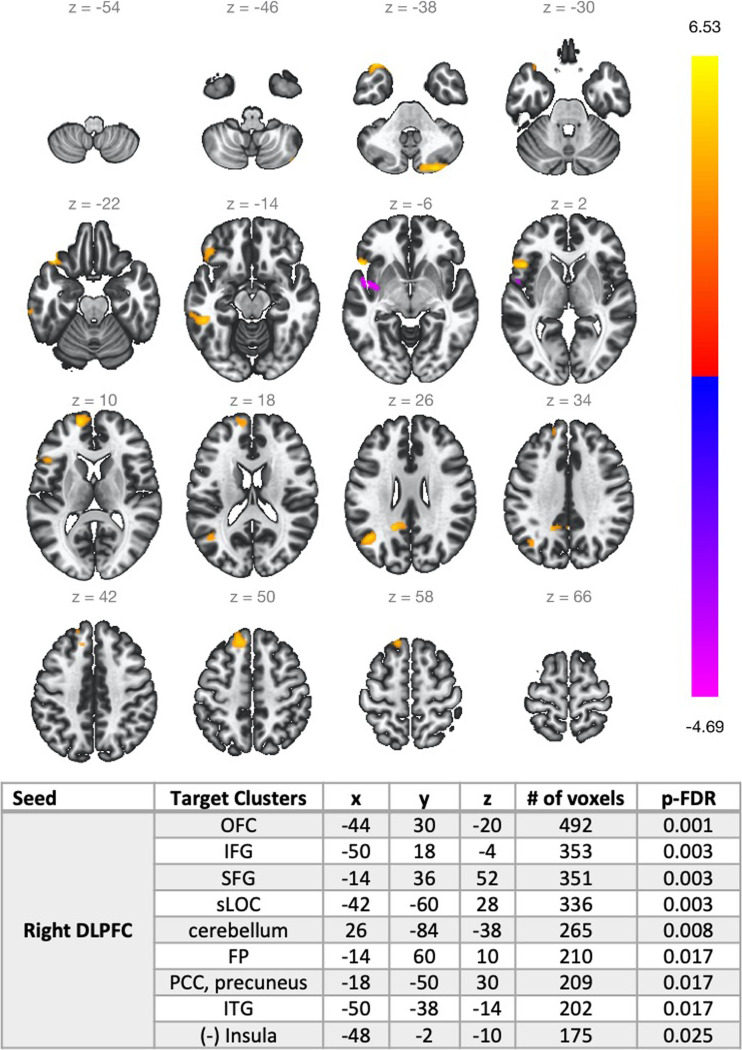
Seed-based whole-brain correlation analysis of changes in rsFC at week 2–3 that were associated with changes in LSAS after week 8 (pre-treatment – week 8). Right DLPFC was used as the seed. Early decreases in rsFC between the right DLPFC-insula, and early increase in rsFC between the right DLPFC-OFC/SFG/ITG, and between the right DLPFC-PCC/precuneus predicted greater improvement in social anxiety at week 8. rsFC, resting state functional connectivity; LSAS, Liebowitz Social Anxiety Scale; OFC, orbitofrontal cortex; DLPFC, dorsolateral prefrontal cortex; IFG, inferior frontal gyrus; SFG, superior frontal gyrus; sLOC, Lateral Occipital Cortex, superior division; FP, frontal pole; PCC, posterior cingulate cortex; ITG, inferior temporal gyrus.

In SAD participants, MVPA analysis revealed two significant clusters, located in the left dorsolateral prefrontal cortex (DLPFC) [−32, 48, 34] and the right DLPFC [28, 54, 26], with rsFC changes at week 2–3 that predicted change in social anxiety (ΔLSAS at week 8). Spatial localizations of the two brain clusters (left and right DLPFC) were within the ECN (see online Supplementary Fig. S3). Early decreases in rsFC between the left DLPFC-rostral anterior cingulate cortex and right DLPFC-insula, respectively (ECN-SN) predicted greater improvement in social anxiety at week 8 (Figs 1 and 2). Early increases within the right DLPFC-OFC/SFG/ITG (within ECN), and between the right DLPFC-PCC/precuneus (ECN-DMN) predicted greater improvement in social anxiety at week 8 (Fig. 2). Controlling for level of depression or presence of concomitant medication did not change the findings (see online Supplementary Figs S6 and S7).

Post-hoc tests assessed whether the rsFC changes that predicted LSAS reduction at week 8 were also associated with early changes in LSAS at week 2–3 (i.e. concurrently with fMRI at early treatment). The whole-brain connectivity maps with two seeds located in the left and right DLPFC were not significantly associated with the LSAS outcome at week 2–3.

- In depression symptoms measured by HAM-D

In SAD participants, MVPA analysis revealed three significant clusters at week 2–3 that predicted change in depression (ΔHAM-D at week 8), located in the superior lateral occipital cortex (sLOC) [34, −66, 24], and posterior inferior temporal gyrus (pITG) (left: [−62, −34, −30] and right [56, −18, −34]). The spatial localization of the three brain clusters (sLOC within visual network, and bilateral pITG within ECN) is displayed in online Supplementary Fig. S8. Greater improvement in depression at week 8 was predicted by early decrease in rsFC between the left pITG and insula and between the right pITG and ACC (ECN-SN), and early increase in rsFC of bilateral pITG-PFC (within ECN) (online Supplementary Fig. S9). No significant associations were found with the sLOC seed region.

Post hoc tests assessed whether ΔrsFC was associated with ΔHAM-D assessed concurrently with MRI at week 2–3. For both seed regions, no significant association was found with HAM-D outcome at week 2–3.

- In social anhedonia symptom measured by the RSAS

The MVPA analysis did not reveal any significant clusters of change in rsFC that were associated with change in RSAS at early treatment or week 8.

#### Early changes in functional connectivity associated with GC-MRT

**Fig. 3.**
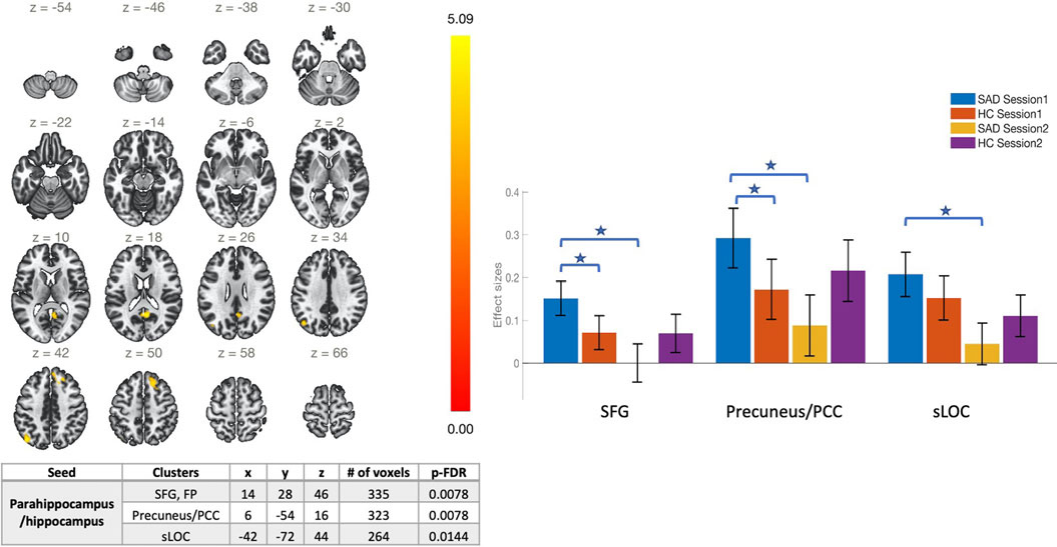
For parahippocampus/hippocampus (HIP) seed region, connectivity decreased significantly from pre- to early-treatment within the DMN (HIP-PCC/precuneus), and between HIP and SFG. HIP, hippocampus; SFG, superior frontal gyrus; PCC, posterior cingulate cortex; sLOC, lateral occipital cortex, superior division; *x y z* are MNI coordinates; DMN, default mode network.

In SAD participants, MVPA analysis of change in rsFC from pre-treatment to week 2–3 revealed one significant cluster in the parahippocampus and HIP (online Supplementary Fig. S5). Using this cluster, a seed-based whole-brain correlation analysis of rs-fMRI data, corrected for multiple comparisons at cluster level (peak level: *p* < 0.001, uncorrected; cluster level: *p* < 0.05, FDR-corrected), showed significant decreases in connectivity within the DMN. Specifically, from pre-treatment to week 2–3, there were significant decreases in rsFC (ΔrsFC) of HIP with PCC/precuneus, SFG, and sLOC (Fig. 3). In HC, MVPA analysis revealed no significant clusters from pre-treatment to week 2–3. Post-hoc analysis also revealed significantly higher within-DMN connectivity (HIP-PCC/precuneus, HIP-SFG) at pre-treatment in SAD, compared with HC. No significant differences were found between HC and SAD at week 2–3 (Fig. 3).

To understand whether these early changes in rsFC were associated with changes in clinical symptoms in SAD group, post-hoc tests assessed the association between ΔrsFC in these three pathways and changes in symptom scores (ΔLSAS, ΔHAM-D) concurrently at week 2–3, and later at week 8. No significant associations were found with clinical symptom change at either time point.

#### Pre-treatment rsFC predictive of improvement in social anxiety and depression

The MVPA analysis did not reveal any significant clusters of pre-treatment rsFC that were associated with change in social anxiety or depression at week 8.

## Discussion

GC-MRT is a novel computer-based treatment that has been shown to reduce attention to socially threatening stimuli and to improve clinical symptoms in patients with SAD (Lazarov et al., 2017), yet its neural mechanisms of action have not been previously studied. This study used resting state fMRI to investigate changes in intrinsic whole-brain functional network connectivity associated with GC-MRT, and to explore their relationship with improvement in clinical symptoms during treatment. Social anxiety, depression, and social anhedonia all improved significantly from pre- to post-treatment in SAD individuals. We identified early changes in brain rsFC (decreased ECN-SN connectivity, and increased connectivity within the ECN, and between ECN and the DMN) that were associated with greater improvement in social anxiety at post-treatment (week 8) and early changes in rsFC within ECN and ECN-SN that were associated with improvement in depression. We also found significantly decreased connectivity within the DMN after 2–3 weeks of treatment in SAD group, while no changes were found in HC over the same time interval. Taken together, these findings suggest that connectivity changes within the ECN and ECN-DMN and ECN-SN may be related to mechanisms of the clinical effects of GC-MRT, warranting further research in randomized controlled trials.

Early changes in rsFC that were associated with improvement in social anxiety included increases within the ECN (right DLPFC-OFC/SFG). Our finding of an early increase in rsFC within the ECN predicting improvement in social anxiety and depressive symptoms is consistent with GC-MRT potentially enhancing attention control, and rsFC may be more sensitive in this regard than our self-report measure of attention control, which did not change during treatment. The increase of rsFC within ECN at this early stage of treatment, at which time improvement in the primary clinical outcome (LSAS) was modest (though statistically significant), suggests that rsFC changes might precede symptomatic response, though this would need to be validated by repeated assessment of rsFC and symptoms at additional time points. Moreover, previous studies have shown that decreased within-ECN connectivity correlated with symptom severity in major depressive disorder (Pan et al., 2020). These results are consistent with our finding that an early increase of rsFC within ECN predicted improvement in depression symptoms.

In addition, we found that an early decrease in ECN-SN rsFC predicted improvement in both social anxiety and depression. The early decrease in ECN-SN rsFC may reflect reduced sensitivity to salience and enhanced cognitive control, which might in turn reduce anxiety and depression (Menon & Uddin, 2010). Compared with HC, SAD patients have been found to exhibit increased connectivity within the SN, possibly related to hypervigilance to external stimuli (Northoff, 2020; Straube, Schmidt, Weiss, Mentzel, & Miltner, 2009). Studies have inconsistent findings on changes of SN and ECN in GAD, SAD, and depression, for example, some studies found decreased connectivity between key nodes in the SN and the ECN in GAD (Etkin, Prater, Schatzberg, Menon, & Greicius, 2009; Kim, Gee, Loucks, Davis, & Whalen, 2011), SAD (Liao et al., 2010b), and depression (Ellard et al., 2018), while other studies found increased rsFC of SN to ECN in depression (Liu et al., 2021) and this increased ECN-SN was correlated with overall depression severity (Li et al., 2017).

We also found that an early increase in ECN-DMN (right DLPFC-PCC/precuneus) rsFC predicted improvement specifically in social anxiety, but not depression. Prior work has shown that better cognitive performance (as measured by working memory and attention tasks) is associated with greater connectivity between the ECN and the DMN, suggesting that these functions may depend upon coupling of these networks (Albert, Potter, Boyd, Kang, & Taylor, 2019; Liang, Zou, He, & Yang, 2016). Moreover, aberrant ECN-DMN connectivity may reflect ongoing rumination, or an underlying bias for control systems to allocate resources toward internal thoughts at the cost of engaging with the external world (Kaiser, Andrews-Hanna, Wager, & Pizzagalli, 2015). Decreased ECN-DMN has been reported in anxiety disorders (Xu et al., 2019). The increased ECN-DMN rsFC we found in SAD may reflect improvement in cognitive functioning, better executive control related to mind-wandering or worry, and less rumination (Sylvester et al., 2012). Finally, the association between increased ECN-DMN rsFC and change in social anxiety but not depression may reflect either true specificity or only that GC-MRT exerted larger effects on social anxiety than depression in our study.

Interestingly, our findings of early changes in brain rsFC (decreased ECN-SN connectivity, and increased connectivity within the ECN, and between ECN and the DMN) that were associated with greater improvement in social anxiety at week 8 were not consistent with findings of previous studies of CBT treatment in SAD (Choi et al., 2016; Prater et al., 2013; Whitfield-Gabrieli et al., 2016). Our study differed in utilizing GC-MRT and in assessing brain changes occurring early in treatment rather than using pre-treatment rsFC to predict treatment outcomes. Additionally, we used a data-driven approach examining the rsFC of all voxels in the brain without an *a priori* hypothesis of a seed region, while most studies prespecified amygdala as the seed region.

Finally, we found significantly higher connectivity within the DMN at pre-treatment in SAD compared with HC, while after 2–3 weeks of treatment there was decreased connectivity within the DMN in SAD group and no changes were found in HC over the same time interval. One previous study also showed increased DMN activity in socially anxious individuals during reward processing, consistent with high self-focused attention even while engaging with potentially rewarding stimuli (Maresh, Allen, & Coan, 2014). A recent review, however, reported hypoconnectivity within DMN in patients with SAD (Lucherini Angeletti et al., 2021). The discrepancy may relate to the review's focus on cortical regions and exclusion of the HIP from the posterior DMN. Many other studies have included the HIP as part of the posterior DMN (Mulders, van Eijndhoven, Schene, Beckmann, & Tendolkar, 2015), as we did. The posterior DMN is implicated in episodic memory retrieval through its connection with temporal structures including the HIP (Andrews-Hanna, Reidler, Huang, & Buckner, 2010). The HIP is an important brain region implicated in both general anxiety and fear memory.

Our finding that DMN connectivity (HIP-PCC/precuneus/FP) decreased significantly after 2–3 weeks of GC-MRT treatment in patients with SAD suggests that GC-MRT may have early effects on DMN rsFC, and normalized it to the levels of HC. This may relate to the treatment's effect of redirecting attention away from social threat stimuli that activate self-focused attention, or alternatively to its more general effect of encouraging focused attention on external stimuli. This may also reflect a treatment effect of less social threat memory retrieval via HIP connectivity. However, these early FC changes were not associated with symptom changes at either early/during treatment or post-treatment, suggesting that other neural processes that are not captured by resting state measures may be more closely related to symptom change. For example, other studies have shown SAD symptoms were associated with brain activity in fear, reward, attention, or executive control circuitry (Crane, Chang, Kinney, & Klumpp, 2021; Mizzi, Pedersen, Lorenzetti, Heinrichs, & Labuschagne, 2021). Further studies could assess other downstream processes by evaluating pre- to post-treatment fMRI scan, which may be more closely related to symptom change.

This study has several limitations. A key limitation is the absence of a non-GC-MRT SAD group to control for non-specific changes in rsFC and clinical symptoms over time. The current findings will need to be confirmed in a randomized controlled trial. Additionally, while the week 2–3 assessment of rsFC was designed to detect early brain changes that might precede clinical improvement, improvement in the primary outcome was already statistically significant at week 3, though improvement became much greater at post-treatment. We cannot preclude the possibility that neural changes during the first 2–3 weeks may be related to concurrent symptom change and non-specific factors. A more fine-grained repeated assessment using rsFC would be needed to clarify the time course of change in rsFC and clinical outcomes. Moreover, we used group-level univariate analysis with relatively small samples and 10 min resting state scans in this study, thus we cannot make individualized prediction of treatment outcome. Further studies with larger samples and longer scans could use multivariate analysis/machine learning algorithms to handle multidimensional imaging data and predict treatment outcome at the individual level. Although this study found that extending GCMRT duration to 12 sessions did not improve clinical outcome, future research could examine other approaches to optimizing GCMRT, such as by increasing the number of faces (more or less than 16) and the presentation time (24 s per matrix). Finally, further research could collect additional behavioral measures of attention control to better understand the scope of clinical correlates of neural changes associated with GC-MRT.

## Conclusion

This initial attempt to identify early neural predictors of response to GC-MRT treatment found that early changes in rsFC within the ECN, between the ECN and DMN, and between the ECN and SN predicted clinical outcome at post-treatment. Consistent with the aim of GC-MRT to target biased attention to threat, these networks are known to play important roles in attention allocation, salience processing and cognitive control. Our preliminary findings support the value of future controlled trials utilizing rsFC to understand profiles of neural networks involved in the engagement and therapeutic action of GC-MRT in SAD.

## Supplementary material

For supplementary material accompanying this paper visit https://doi.org/10.1017/S0033291721005171. click here to view supplementary material
