# Supplementary material for: Resting state connectivity predictors of symptom change during gaze-contingent music reward therapy of social anxiety disorder
Source: Psychol Med. 2022 Mar 22;53(7):3115–23. doi: 10.1017/S0033291721005171 (PMC9612546; doi:10.1017/S0033291721005171)
Supplement: Supplementary file 1 [file S0033291721005171sup001.docx]

**Supplementary Material:**

**Participants**

Participants were recruited through clinical referrals and online advertisements. After a telephone screen to assess eligibility, potential participants completed the self-rated version of the Liebowitz Social Anxiety Scale (LSAS-SR) (Fresco et al., 2001), and underwent a clinical assessment by a psychiatrist. Primary and co-morbid diagnoses were ascertained using the Mini-International Neuropsychiatric Interview version 7.0.2 (MINI) (Sheehan et al., 1998). Each participant was assessed by a single psychiatrist, and a total of three psychiatrists conducted assessments for the study. Reliability was maintained through shared training and co-rating a subset of cases. Interrater reliability for the primary clinical outcome measure, the LSAS, was excellent (*r* > 0.90).

**Inclusion criteria**

Inclusion criteria for SAD participants were: a) primary DSM-5 diagnosis of SAD; b) LSAS score > 50; c) 18-60 years old; d) fluent English; and e) normal or corrected-to-normal vision. Key exclusion criteria included a current severe depression indicated by a Hamilton Rating Scale for Depression (HAM-D) 17-item score > 20 (Hamilton, 1960). Severe depression was exclusionary because exposing persons with severe depression to 8 weeks of an experimental treatment directed primarily at social anxiety might be considered unethical. Additionally, as social anxiety disorder was required to be the principal diagnosis, this would be incompatible with a comorbid diagnosis of severe depression. Additional exclusion criteria included: a) clinically significant suicidal ideation or behavior; b) current or past psychosis; c) current or past diagnosis of PTSD, obsessive-compulsive disorder, bipolar disorder, manic episode, tic disorder, or ADHD; d) severe alcohol or cannabis use disorder, and/or any severity of other substance use disorder (except nicotine use disorders); e) current unstable or untreated medical illness; f) current or neurocognitive disorder, seizure disorder or brain injury; g) use of any psychotropic medication in the past month, other than a serotonin reuptake inhibitor, serotonin-norepinephrine reuptake inhibitor, or zolpidem for sleep, that had been taken at a stable dose for at least three months (see supplement for full criteria); h) any concurrent cognitive behavioral therapy (CBT), or other psychotherapy that was initiated within the past three months; i) eye-tracking calibration difficulties; or j) having contraindications to MRI. HC participants were age- and sex-matched, and did not have any lifetime psychiatric disorders or current psychiatric medication use. All participants were right-handed.

**Randomization**

Participants were randomized to a treatment duration of either 4 weeks or 8 weeks, with randomization stratified by presence or absence of pre-existing medication treatment. The randomization schedule was developed using a web-based randomizer (<https://www.randomizer.org>) to create blocks of 10 randomized sets ranging from 1 to 4 where even numbers reflected the 4 week condition and odd numbers indicated the 8 week condition. Subjects were assigned randomized slots in the order that they enrolled in the study.

**Measures**

**Liebowitz Social Anxiety Scale (LSAS) (Liebowitz, 1987).** The LSAS is a 48-item measure rating fear and avoidance of 24 social situations on a scale from 0 (none) to 3 (severe). The self-rated version was used as a screener, and the clinician-administered version was the primary clinical outcome for the current study.

**Hamilton Rating Scale for Depression (HAM-D) (Hamilton, 1960).** The 17-item version of the HAM-D was used to assess depression symptoms.

**Revised Social Anhedonia Scale (RSAS) (Cicero, Krieg, Becker, & Kerns, 2016).** The RSAS is a 40-item self-report scale that assesses anhedonia relating to social situations and stimuli. Participants read 40 statements about their enjoyment of social situations and rate “yes” or “no” to indicate whether they agree or disagree with each statement.

**Snaith-Hamilton Pleasure Scale (SHAPS) (Snaith et al., 1995).** The SHAPS is a 14-item self-report measure of anhedonia. Participants rate how much they agree or disagree with statements related to enjoying typically pleasurable situations (e.g., “I would enjoy being with my family or close friends”, “I would enjoy my favorite meal”) on a scale from 1 (Definitely Agree) to 4 (Definitely Disagree). Higher scores indicate greater anhedonia. This measure was scored by totaling all item scores (Franken, Rassin, & Muris, 2007).

**Attention Control Scale (ACS) (Derryberry & Reed, 2002).** The ACS is a 20-item self-report scale that measures perceived attentional control. Participants rate statements relating to their abilities to focus attention, shift attention, and control their thoughts on a scale from 1 (almost never) to 4 (always).

**Preprocessing of imaging data**

fMRI images were preprocessed using MATLAB version R2018a (The MathWorks, Inc., Natick, Massachusetts) and statistical parametric mapping software (SPM12; Welcome Trust Centre for Neuroimaging, UCL, London, United Kingdom). Preprocessing steps included slice-time correction and motion correction using a six-parameter rigid body transformation, then co-registration to each participant’s T1-weighted structural image. Co-registered images were normalized to the Montreal Neurological Institute (MNI) canonical template, and smoothed with an 8mm full-width-at-half-maximum (FWHM) Gaussian kernel. Functional connectivity analyses were performed on the smoothed images.

**Functional connectivity analysis**

For each subject and session, the MVPA method computed pair-wise correlations between each voxel to all other voxels. Principal components analysis (PCA) was used to reduce data dimensionality (default number of 64 components) by projecting the data from high dimensional space to lower dimensional subspace (principal subspace) such that the variance of the projected data was maximized. The four strongest spatial principal components were selected based on an approximate 5:1 ratio between observations (N=20 for HC, N=33 for SAD) and independent variables (Byun et al., 2021; Thompson, Thelin, Lilja, Bellander, & Fransson, 2016). For a given voxel, *vi*, a PCA was performed by retaining 4 components, across all subjects, between *vi*'s connectivity with all other voxels. In other words, each voxel had a 4-dimensional representation of the spatial pattern of its connectivity to all other voxels for each subject. Then, to assess the association between changes in rsFC from pre-treatment to week 2-3 or pre-treatment rsFC with changes in SAD symptoms, an omnibus *F* test was carried out comparing the between-subject variance in relation to clinical outcomes in SAD across all voxels' connectivity patterns in 4-dimensional space. Scanner was used as nuisance regressor. This test yielded clusters of voxels (seeds) that displayed a similar between-subject variance of their spatial connectivity. Once seeds were established, the seeds from the MVPA were extracted as region of interest (ROI) masks and were applied for seed-to-voxel whole-brain analysis to determine whole-brain connectivity patterns that were associated with the clinical outcomes.

**Supplemental Results:**

SAD participants were randomized to a treatment duration of either 4 weeks or 8 weeks. To assess whether there was a main effect of treatment groups, a treatment group (4 weeks vs 8 weeks) by time points (wk 0, wk 2-3, wk 8) ANOVA was carried out for the LSAS, HAM-D, RSAS, SHAPS, and ACS. There was no significant treatment group by time interaction or main effect of treatment groups in any clinical measures. A significant main effect of time was found for the LSAS (F=7.15, df=2, p=0.001).

**Supplemental Figures:**

**Figure 1:** Flowchart

## Enrollment

Assessed for eligibility (n=72)

Excluded (n=25)

- Not meeting inclusion criteria (n=16)
  - LSAS < 50 (n=1)
  - HAM-D > 20 (n=1)
- Eligible but not randomized (n=9)
  - COVID-19 suspension (n=2)
  - Scheduling difficulties (n=5)
  - Eye tracking difficulties (n=2)

## Allocation

Randomized (n= 47)

4-week treatment (n=24)

- **Received allocated intervention (n=21)**
- Did not receive allocated intervention (n=3)
  - Lost to followup (n=1)
  - No longer met inclusion criteria (n=1)
  - Discontinued due to scheduling difficulties (n=1)
- **Completed pre-treatment MRI scan (n=21)**
- Did not complete pre-treatment MRI scan (n=3)
  - Discontinued prior to scan (n=3)

8-week treatment (n=23)

- **Received allocated intervention (n= 20)**
- Did not receive allocated intervention (n=3)
  - Lost to follow-up (n=2)
  - Discontinued due to scheduling difficulties (n=1)
- **Completed pre-treatment MRI scan (n=22)**
- Did not complete pre-treatment MRI scan (n=1)
  - Exclusionary metal in body (n=1)
- Completed intervention (n=18)
- Discontinued intervention (n=3)
  - Scheduling difficulties (n=1)
  - Discontinued due to COVID-19 suspension (n=2)
- Completed second MRI scan (n=18)
- Did not complete second MRI scan (n=3)
  - Scheduling difficulties (n=1)
  - Discontinued prior to scan (n=2)
- Completed intervention (n=15)
- Discontinued intervention (n=5)
  - Scheduling difficulties (n=2)
  - Discontinued due to COVID-19 suspension (n=3)
- Completed second MRI scan (n=15)
- Did not complete second MRI scan (n=7)
  - Exclusionary metal in body (n=1)
  - Scheduling difficulties (n=1)
  - Discontinued prior to scan (n=5)

## Intervention

Analyzed (n=18)

Excluded from analysis (n=3)

- Did not receive second MRI scan (n=3)

Analyzed (n=15)

Excluded from analysis (n=7)

- Did not complete any MRI scans (n=1)
- Did not receive second MRI scan (n=6)

## Analysis

*
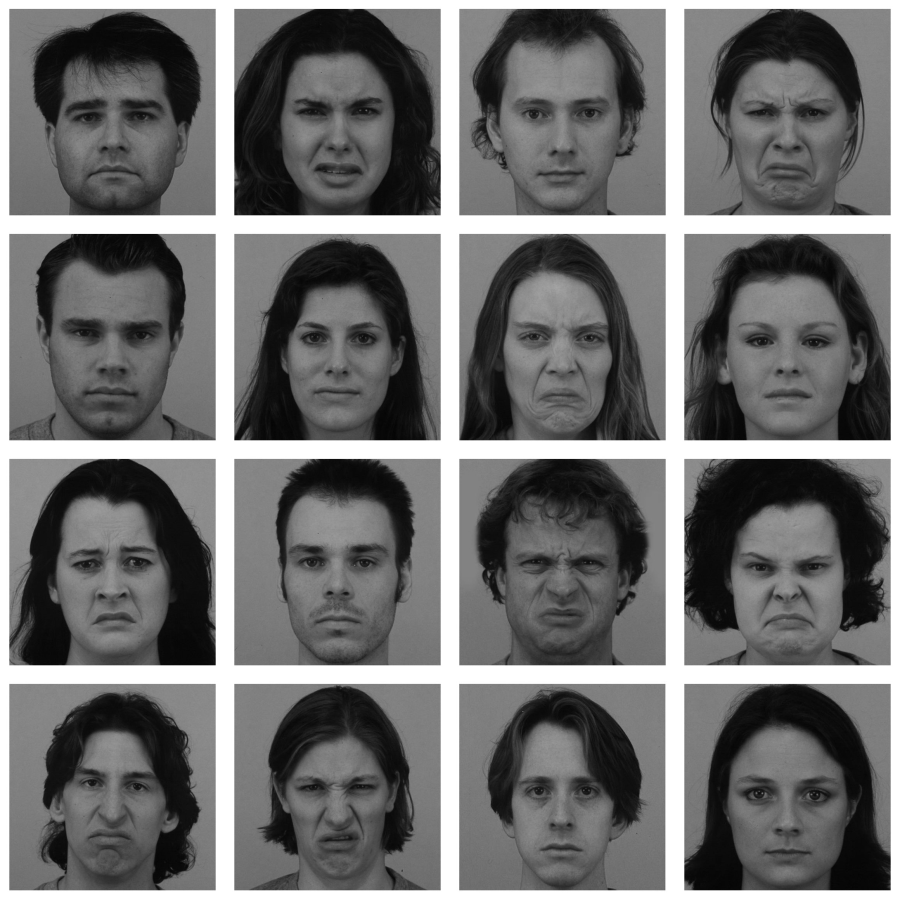
*

**Supplemental Figure 2.** An example of a single matrix. The eight disgusted faces comprise the threat area of interest (AOI) and the eight neutral faces comprise the neutral AOI.


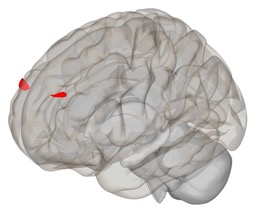

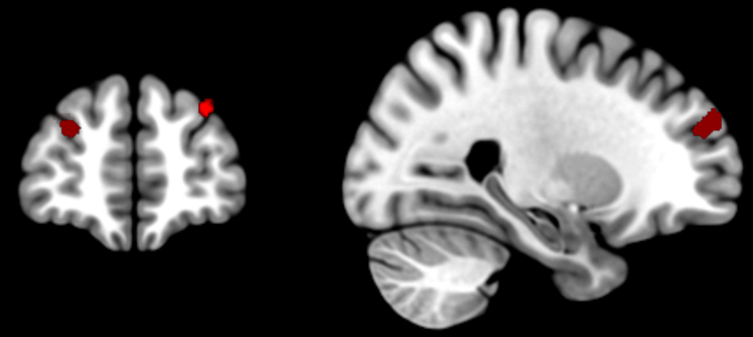

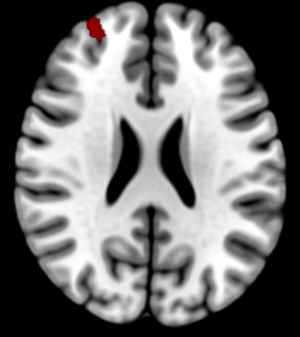

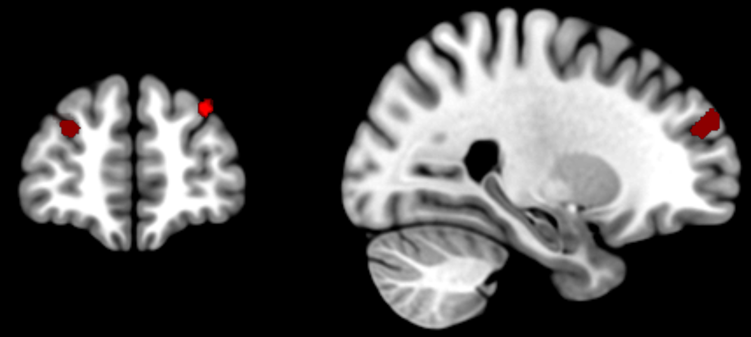


Left DLPFC

Right DLPFC

**Supp****lemental Figure 3**: Localization of the two brain clusters (Left DLPFC [-32 48 34] and right DLPFC [28 54 26]) identified using MVPA where rsFC changes at week 2-3 were associated with clinical outcome after week 8 (LSAS). LSAS: Liebowitz Social Anxiety Scale; DLPFC: dorsolateral prefrontal cortex;


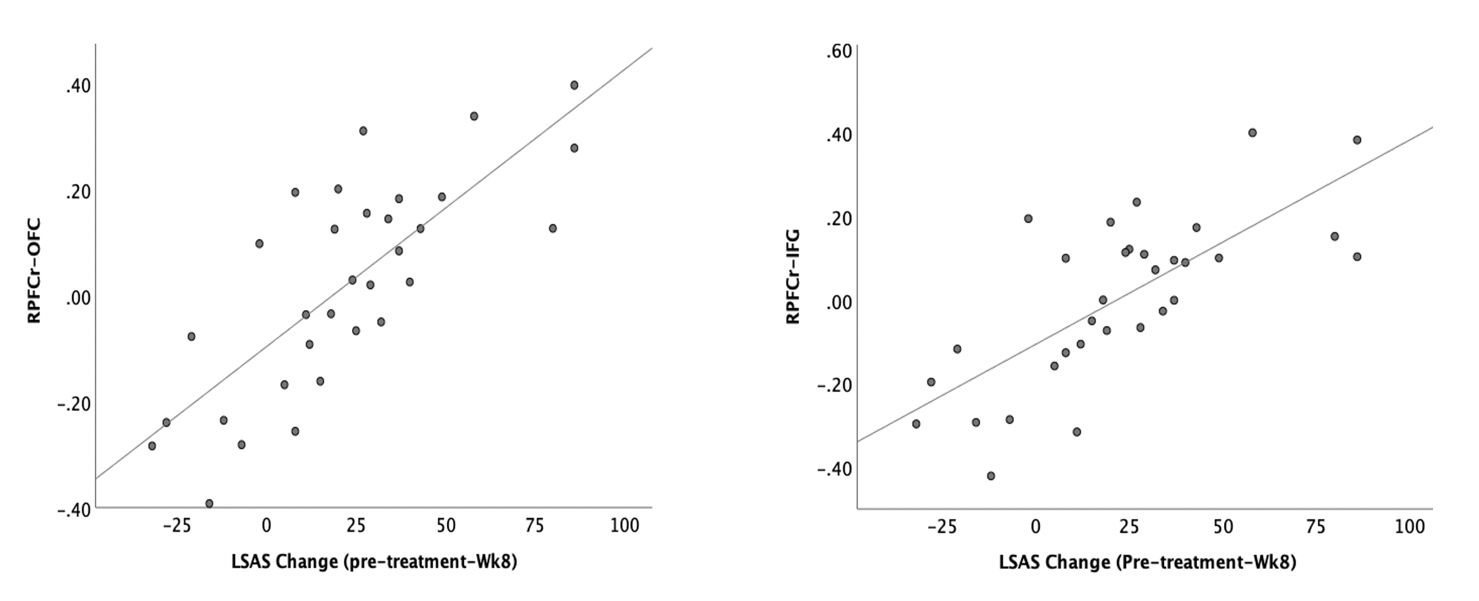


**Supplemental Figure 4:** Correlation between the pathways identified in Figure 3 (right DLPFC-OFC r=0.78 p<0.001, right DLPFC-IFG r=0.737, p<0.001). DLPFC: dorsolateral prefrontal cortex; OFC: orbitofrontal cortex; IFG: inferior frontal gyrus


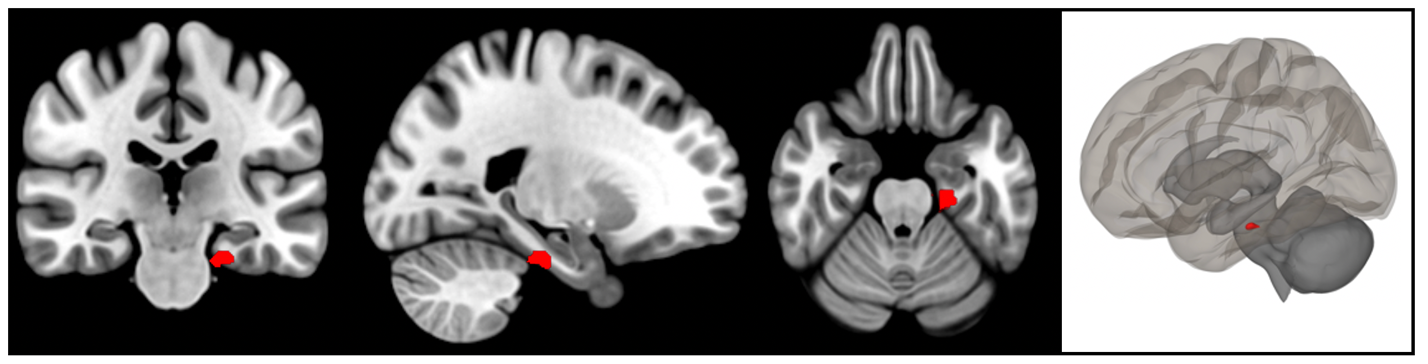


**Supplemental Figure 5**: The MVPA analysis of change from pre-treatment to week 2-3 in the SAD group revealed one significant cluster located in the parahippocampus/hippocampus [-20 -24 -26], which was used as the seed for further whole brain connectivity analysis.


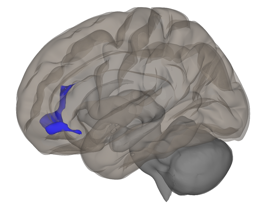

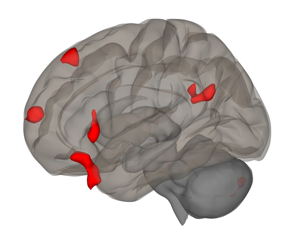


|  | Clusters | x | y | Z | Size | p-FDR |
| --- | --- | --- | --- | --- | --- | --- |
| Left DLPFC | (-) ACC | 10 | 40 | -6 | 851 | <0.001 |
| Right DLPFC | OFC | -44 | 30 | -20 | 468 | 0.0014 |
|  | AG/PCC | -18 | -50 | 30 | 393 | 0.0023 |
|  | Left DLPFC | -16 | 60 | 10 | 262 | 0.0128 |
|  | IFG/OFG | -50 | 20 | -4 | 249 | 0.0128 |
|  | cerebellum | 26 | -84 | -38 | 228 | 0.0131 |
|  | SFG | -14 | 36 | 54 | 226 | 0.0131 |

**Supplemental Figure 6:** Seed-based whole-brain correlation analysis of changes in rsFC that were associated with changes in LSAS after week 8, controlled for HAM-D (pre-treatment -Week 8). The left image used Left DLPFC as the seed, and the right image used right DLPFC as the seed. Note: DLPFC: dorsolateral prefrontal cortex; ACC: anterior cingulate cortex; OFC: orbitofrontal cortex; IFG: inferior frontal gyrus; SFG: superior frontal gyrus; AG: angular gyrus; PCC: posterior cingulate cortex


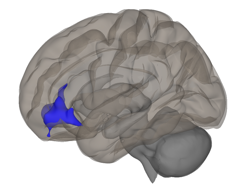

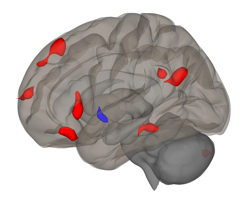


|  | Clusters | x | y | z | Size | p-FDR |
| --- | --- | --- | --- | --- | --- | --- |
| Left DLPFC | (-) ACC | -16 | 44 | -14 | 1585 | <0.001 |
| Right DLPFC | SFG | -14 | 36 | 52 | 346 | 0.006 |
|  | sLOCl/AG | -42 | -60 | 28 | 329 | 0.006 |
|  | IFG | -48 | 20 | -2 | 270 | 0.011 |
|  | FP | -14 | 60 | 10 | 255 | 0.011 |
|  | MTG, ITG | -50 | -38 | -14 | 221 | 0.017 |
|  | OFC | -44 | 28 | -22 | 213 | 0.017 |
|  | Cerebellum | 26 | -84 | -38 | 176 | 0.030 |
|  | PCC | -18 | -50 | 28 | 151 | 0.043 |
|  | (-) Insula | -50 | 4 | -4 | 148 | 0.043 |

**Supplemental Figure 7:** Seed-based whole-brain correlation analysis of changes in rsFC that were associated with changes in LSAS after week 8, controlled for medication (pre-treatment -Week 8). The left image used Left DLPFC as the seed, and the right image used Right DLPFC as seed.

Note: DLPFC: dorsolateral prefrontal cortex; ACC: anterior cingulate cortex: SFG: superior frontal gyrus; sLOC: Lateral Occipital Cortex, superior division, AG: angular gyrus; IFG: inferior frontal gyrus; FP: frontal pole; MTG: Middle Temporal Gyrus; ITG: Inferior Temporal Gyrus; OFC: orbitofrontal cortex; PCC: posterior cingulate cortex.


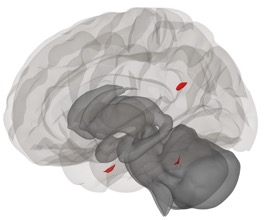


**Supplemental Figure 8:** Spatial localization of the three brain clusters (seeds) in the sLOCr [34 -66 24], right pITG [56 -18 -34] and left pITG [-62 -34 -30] that showed rsFC changes associated with HAM-D change after week 8. Note: sLOC: Lateral Occipital Cortex, superior division; pITG: Inferior Temporal Gyrus, posterior division; rsFC: resting state functional connectivity; HAM-D: Hamilton Rating Scale for Depression


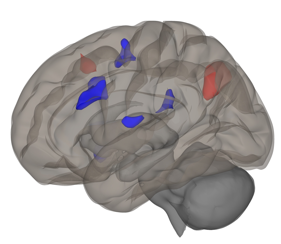

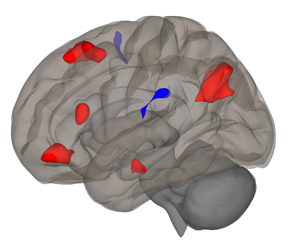


| Seed | Clusters | x | y | z | size | p-FDR |
| --- | --- | --- | --- | --- | --- | --- |
| Left pITG | (-) ACC | -8 | 22 | 24 | 548 | 0.0001 |
|  | (-) Insula | 52 | 8 | -12 | 472 | 0.0003 |
|  | (-) SMA | 6 | 0 | 50 | 267 | 0.0066 |
|  | SFG | 18 | 20 | 48 | 169 | 0.0292 |
|  | sLOC | 38 | -64 | 44 | 621 | 0.0001 |
| Right pITG | sLOC | -32 | -60 | 26 | 632 | 0.0001 |
|  | SFG | -18 | 24 | 58 | 569 | 0.0001 |
|  | OFC, FP | -42 | 38 | -10 | 395 | 0.0011 |
|  | sLOC | 36 | -60 | 38 | 347 | 0.0019 |
|  | (-) Insula | 30 | 16 | 6 | 327 | 0.0021 |
|  | IFG | -50 | 22 | 14 | 238 | 0.0089 |
|  | (-) aSMG | -68 | -28 | 28 | 156 | 0.0415 |
|  | SFG | 22 | 24 | 54 | 136 | 0.0468 |
|  | MTG | -54 | -10 | -18 | 135 | 0.0468 |

**Supplemental Figure 9**: Seed-based whole-brain correlation analysis of changes in rsFC associated with HAM-D changes after week 8. Left image used left pITG [-62 -34 -30] as seed, and the right image used right pITG [56 -18 -34] as seeds. Note: pITG: Inferior Temporal Gyrus, posterior division; SFG: Superior Frontal Gyrus; SMA: Supplementary Motor Cortex; sLOC: Lateral Occipital Cortex, superior division; MTG: Middle Temporal Gyrus; aSMG: Supramarginal Gyrus, anterior division; IFG: Inferior Frontal Gyrus; OFC: orbitofrontal cortex; FP: frontal pole.


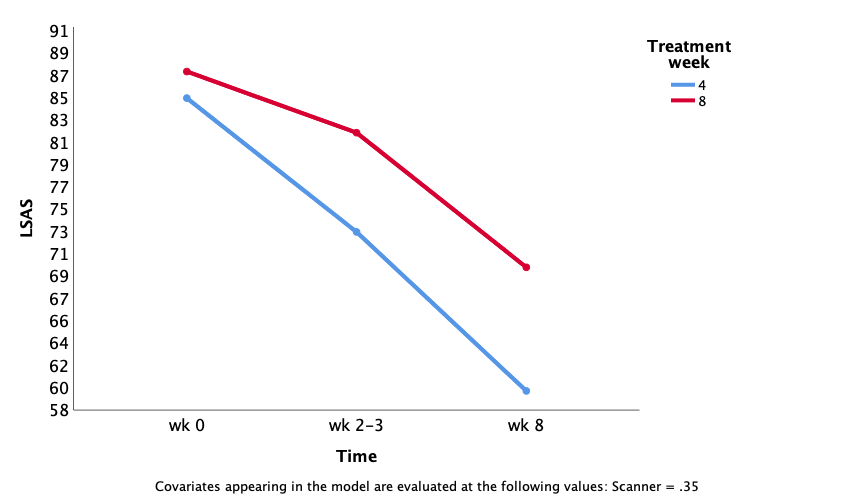

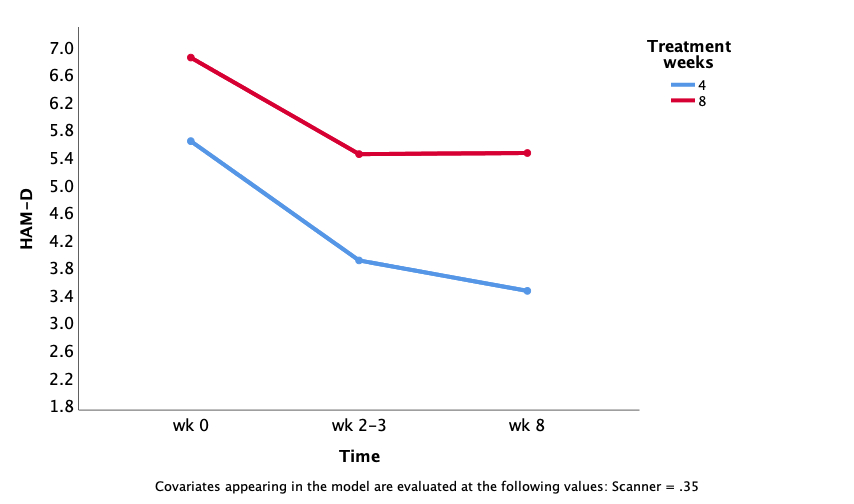


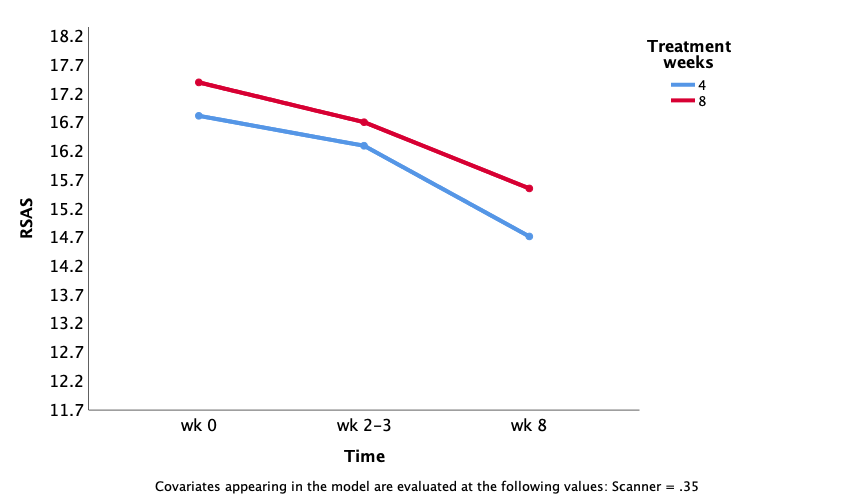

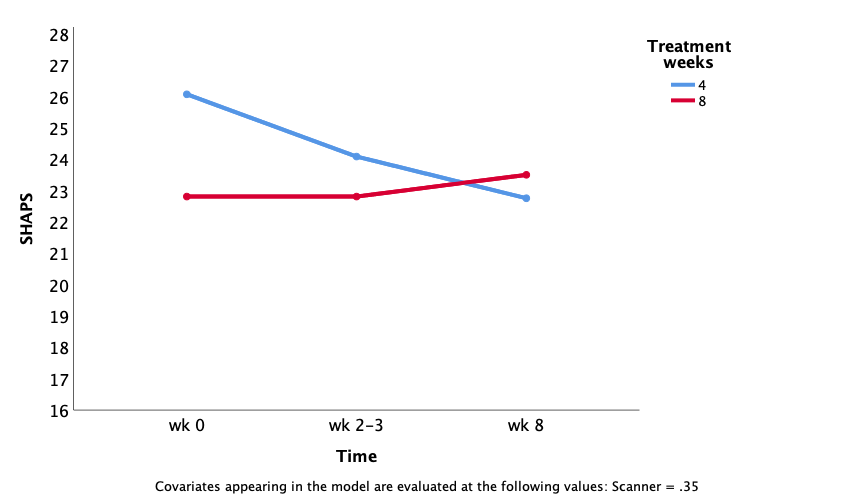


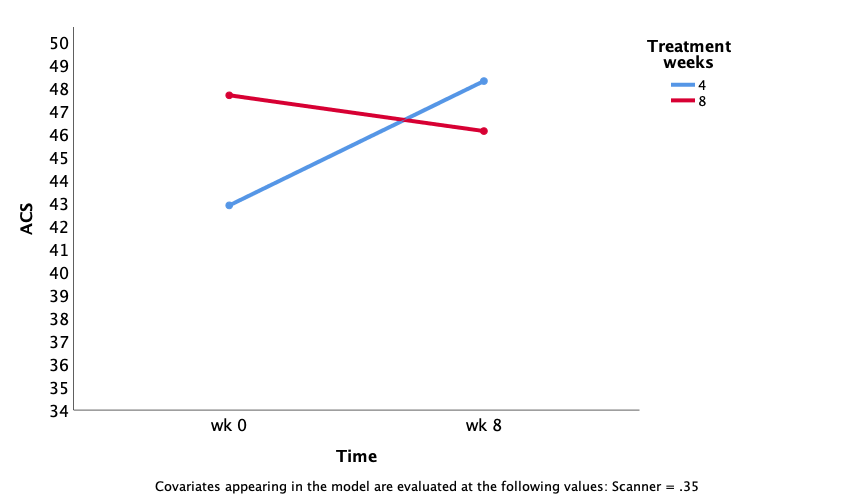


**Supplemental Figure 10:** SAD participants were randomized to a treatment duration of either 4 weeks or 8 weeks. A treatment group (4 weeks vs 8 weeks) by time points (wk 0, wk 2-3, wk 8) ANOVA was carried out for LSAS, HAM-D, RSAS, SHAPS, and ACS.

LSAS: Liebowitz Social Anxiety Scale; HAM-D: Hamilton Rating Scale for Depression; RSAS: Revised Social Anhedonia Scale; SHAPS: Snaith-Hamilton Pleasure Scale; ACS: Attention Control Scale.

**Reference:**

Byun, J. I., Cha, K. S., Kim, M., Lee, W. J., Lee, H. S., Sunwoo, J. S., . . . Jung, K. Y. (2021). Altered insular functional connectivity in isolated REM sleep behavior disorder: a data-driven functional MRI study. *Sleep Medicine, 79*, 88-93. doi:10.1016/j.sleep.2020.12.038

Cicero, D. C., Krieg, A., Becker, T. M., & Kerns, J. G. (2016). Evidence for the Discriminant Validity of the Revised Social Anhedonia Scale From Social Anxiety. *Assessment, 23*(5), 544-556. doi:10.1177/1073191115590851

Derryberry, D., & Reed, M. A. (2002). Anxiety-related attentional biases and their regulation by attentional control. *Journal of Abnormal Psychology, 111*(2), 225-236. doi:10.1037//0021-843x.111.2.225

Franken, I. H., Rassin, E., & Muris, P. (2007). The assessment of anhedonia in clinical and non-clinical populations: further validation of the Snaith-Hamilton Pleasure Scale (SHAPS). *Journal of Affective Disorders, 99*(1-3), 83-89. doi:10.1016/j.jad.2006.08.020

Fresco, D. M., Coles, M. E., Heimberg, R. G., Liebowitz, M. R., Hami, S., Stein, M. B., & Goetz, D. (2001). The Liebowitz Social Anxiety Scale: a comparison of the psychometric properties of self-report and clinician-administered formats. *Psychological Medicine, 31*(6), 1025-1035. doi:10.1017/s0033291701004056

Hamilton, M. (1960). A rating scale for depression. *Journal of Neurology, Neurosurgery, and Psychiatry, 23*, 56-62. doi:10.1136/jnnp.23.1.56

Liebowitz, M. R. (1987). Social phobia. *Modern Problems of Pharmacopsychiatry, 22*, 141-173. doi:10.1159/000414022

Sheehan, D. V., Lecrubier, Y., Sheehan, K. H., Amorim, P., Janavs, J., Weiller, E., . . . Dunbar, G. C. (1998). The Mini-International Neuropsychiatric Interview (M.I.N.I.): the development and validation of a structured diagnostic psychiatric interview for DSM-IV and ICD-10. *Journal of Clinical Psychiatry, 59 Suppl 20*, 22-33;quiz 34-57. Retrieved from https://www.ncbi.nlm.nih.gov/pubmed/9881538

Snaith, R. P., Hamilton, M., Morley, S., Humayan, A., Hargreaves, D., & Trigwell, P. (1995). A scale for the assessment of hedonic tone the Snaith-Hamilton Pleasure Scale. *British Journal of Psychiatry, 167*(1), 99-103. doi:10.1192/bjp.167.1.99

Thompson, W. H., Thelin, E. P., Lilja, A., Bellander, B. M., & Fransson, P. (2016). Functional resting-state fMRI connectivity correlates with serum levels of the S100B protein in the acute phase of traumatic brain injury. *Neuroimage Clinical, 12*, 1004-1012. doi:10.1016/j.nicl.2016.05.005
